# The World’s Columbian Dental Congress

**Published:** 1892-02

**Authors:** 


### The World's Columbian Dental Congress.

A meeting of the Executive Committee of the World's Columbian Dental Meeting (now Dental Congress) was held in Chicago, January 11th and 12th, the object of which was to further provide for that great occasion.

The change of the name from "Meeting" to "Congress" was done by the unanimous vote of the committee at the suggestion of the President of the World's Congress Auxiliary. This he did in order that the name of the dental meeting might be harmonious with that of several other congresses to be held during the time of the World's Fair. The work of the committee consists largely in filling out a number of committees that had already been designated and defining, so far as practicable, the work of these respective committees. Three or four new committees were also designated and appointments made for them.

The time for the meeting of the Dental Congress was fixed for August 17th to 27th, 1893; a change, it will be observed, in this respect from the time formerly suggested.

Ample room and accommodation for all the work connected with this meeting will be provided by the authorities in the Art Palace, the main room of which will accommodate about thirty-five hundred; there will be a large number of smaller rooms for clinics, committees, sections, etc. Several of the committees have been vigorously at work for a number of months past; nearly all will from this time on be actively engaged in their special work.

There was in this meeting of the executive committee, in every thing done by them the utmost harmony, and an evident determination on the part of every one to do every thing possible for the highest success of this great meeting, and in the profession throughout the country there is an enthusiasm in reference to this matter that could hardly have been anticipated by anyone.

We hoped to have had for publication in the present number a full minute of the proceedings of this meeting of the committee, but it is not yet at hand, and must be deferred till the next number.

To Obtain Pure Oxygen Rapidly.—Zinno's method consists in mixing intimately 200 gm. of powdered potassium permanganate with an equal weight of barium binoxide. On the addition of water oxygen is disengaged. With the amount stated, at ordinary temperature, 13,620 cc. of pure oxyen are generated. The oxygen is rapidly produced, and is not contaminated by chlorine, or chlorine products.—*Med. and Surg. Rept.*

